# Gynecologic Oncology Sub-Specialty Training in Ghana: A Model for Sustainable Impact on Gynecologic Cancer Care in Sub-Saharan Africa

**DOI:** 10.3389/fpubh.2020.603391

**Published:** 2020-12-03

**Authors:** Anna Sarah Erem, Adu Appiah-Kubi, Thomas Okpoti Konney, Kwabena Amo-Antwi, Sarah G. Bell, Timothy R. B. Johnson, Carolyn Johnston, Alexander Tawiah Odoi, Emma R. Lawrence

**Affiliations:** ^1^Saba University School of Medicine, The Bottom, Saba, Netherlands; ^2^Department of Obstetrics and Gynaecology, University of Health and Allied Sciences, Ho, Ghana; ^3^Gynaecologic Oncology Unit, Department of Obstetrics and Gynaecology, Komfo Anokye Teaching Hospital (KATH), Kumasi, Ghana; ^4^Department of Obstetrics and Gynaecology, School of Medicine and Dentistry, Kwame Nkrumah University of Science and Technology, Kumasi, Ghana; ^5^Department of Obstetrics and Gynecology, University of Michigan, Ann Arbor, MI, United States; ^6^Division of Gynecologic Oncology, Department of Obstetrics and Gynecology, University of Michigan, Ann Arbor, MI, United States

**Keywords:** medical education, trends and challenges, twenty-first century, gynecologic oncology fellowship, Ghana

## Abstract

African women have double the risk of dying from cancer than women in high-income countries. In Ghana, most women with gynecological malignancies present with advanced-stage disease when treatment is less effective. Barriers to improved cancer outcomes include the availability of cancer screening, affordability of treatment, and access to gynecologic oncology specialists. In response to a paucity of gynecologic oncology providers, an in-country fellowship training program was established at Komfo Anokye Teaching Hospital (KATH) in 2013. Historically, Ghanaian resident physicians were sent to other countries for fellowship training and were unlikely to repatriate. The establishment of an in-country training program not only addresses the challenge of “brain drain,” but also builds local capacity in gynecologic oncology education and emphasizes culturally relevant and accessible healthcare. The four-years gynecologic oncology fellowship program at KATH was developed as part of a longitudinal multi-decade partnership between the University of Michigan and academic medical centers in Ghana. The fellowship trains obstetricians and gynecologists to provide subspecialist clinical and surgical care to patients with gynecologic malignancies. Fellows collaborate with the radiation, oncology and pathology departments, participate in monthly inter-institutional tumor board meetings, conduct research, advise on health policy issues, and train subsequent cohorts. This fellowship is representative of emerging twenty-first-century trends in which subspecialty training programs in low-income countries are strengthened by international collaborations. Providing specialized training in gynecologic oncology can help develop and maintain resources that will improve clinical outcomes for women in low-resources settings.

## Introduction

Across Sub-Saharan Africa (SSA), the prevalence of gynecological malignancies is high, and subsequent morbidity and mortality are significant. In particular, cervical cancer is uniquely problematic in low-resource settings where access to routine screening is minimal. Gynecologic malignancies disproportionately affect reproductive age women, resulting in economic and social consequences for women, their families, and their communities. Due to the complexities of medical and surgical management of gynecologic malignancies, routine medical training is not sufficient to provide this specialized care. A lack of trained gynecologic oncology specialists contributes to poor patient outcomes, with a critical need for specialists to lead diagnosis, imaging, and surgical and oncologic care. Local training programs in gynecologic oncology also contribute to cancer screening initiatives, capacity for chemotherapy and radiation, and locally driven research.

We stress the importance of global partnerships in high-impact subspecialized care like gynecologic oncology. These partnerships utilize existing infrastructure and relationships to access funding and build healthcare and research capacity in low-resource settings. We explore the structure and commonalities of existing gynecologic oncology training programs across Africa, including South Africa, Zambia, Uganda, Kenya, Ethiopia, Mozambique, and Ghana.

We discuss in detail a gynecologic oncology fellowship program at the Komfo Anokye Teaching Hospital (KATH) in Ghana established in 2013 through a partnership between the University of Michigan and the Ghana College of Physicians and Surgeons (GCPS). We outline the structure of the gynecologic oncology fellowship program, including goals, entrance requirements, didactics, research requirements, evaluation processes, and local and external faculty support. Finally, to gain a personal perspective on gynecologic oncology fellowship training, we explore responses to semi-structured interviews completed by Ghanaian fellows.

## Prevalence of Gynecological Malignancies in Sub-Saharan Africa and Ghana

In Sub-Saharan Africa (SSA), cancer has historically taken a back seat to communicable diseases. Now that treatment of communicable diseases has improved, and life expectancies have increased, a large focus of healthcare has shifted to treatment of chronic diseases, including cancer (1). In 2018, cervical cancer was the leading cancer in half of the countries in SSA and responsible for 21.7% of all cancer deaths in SSA women (2). While the probability of developing cancer for a woman living in Uganda or Zimbabwe is 30% lower than a Western European woman, her probability of death is almost twice as high (3). In low- and middle-income (LMIC) families, women play many essential roles, including providing food, and contributing to economic support (4–6). Thus, morbidity and mortality secondary to gynecologic malignancies is especially devastating.

Demographic and health infrastructure factors unique to SSA drive the epidemiology of gynecologic cancer in the region. In SSA, higher parity and lower incidence of obesity are protective against endometrial cancer (7). However, recent trends in industrialization and urbanization, changes in smoking and alcohol consumption, increasing obesity rates, and decreased parity may increase cancer risk (8, 9). In SSA, factors contributing to the delays in diagnosis and treatment of gynecological cancers include a lack of knowledge among women, lack of confidence in a cure, fear of the cost of treatment, travel time to a treatment center, and concern over being outcast by their family (10).

Unlike high-income countries, cervical cancer is a leading cause of cancer burden and death in LMIC (11, 12), including in the West African country of Ghana (8). The annualized age-standardized incidence rate of cervical cancer in Ghana is 32.9 per 100,000 people, four times the incidence seen in high-income countries (13). Barriers to cervical cancer screening, low health literacy, and limited capacity for treatment contribute to higher incidence and morbidity of cervical cancer in Ghana. A review of the cancer registry at Korle Bu Teaching Hospital, a major cancer referral center in Ghana, found that the majority of cervical cancers were diagnosed at a late stage, and more than fifty percent of the cancers were not resectable at diagnosis (8). More than half of Ghanaian women with significant risk factors, including multiple partners and early age at first intercourse, were unaware that they were at risk for cervical cancer (14).

## Relevance of Oncological Specialties in Sub-Saharan Africa

One of the essential components of gynecological cancer diagnoses and management is healthcare staff specially trained in gynecologic cancer care, including gynecologic surgeons, medical oncologists, and radiation oncologists (10). Several studies indicate that the outcomes of patients with gynecologic cancers in high-income countries are improved when they receive treatment from a gynecologic oncologist (15–18). In LMICs, poor patient outcomes and low cure rates are partially attributed to the scarcity of trained gynecologic oncology surgeons (6). In SSA, local healthcare providers and medical facilities capable of managing cancer are woefully insufficient. The number of oncologists per country in Africa ranges from zero in Togo, Chad, and Burundi, to 1,500 in Egypt (19). Ghana has only four clinical oncologists (19). The workload is daunting with 25 countries in Africa reporting an incidence of cancer >1,000 per oncologist (19).

Gynecologists with subspecialty training in oncology can perform complex pelvic surgeries, allowing for surgical resection and debulking, with improvement in prognosis (6). Also key in the delivery of care for women with gynecological cancers in SSA is access to radiation oncologists and radiation facilities. Radiation therapy is an essential mode of therapy of at least half of all cancers in Africa (20). The International Atomic Energy Commission recommends a radiotherapy facility for every 250,000–500,000 people, but in SSA there are 140 facilities for a population of over 1.2 billion (21). Ghana, a country with a population of 30 million people, has only three radiotherapy facilities, 12 radiation oncologists, and ten resident radiation oncologists in training (22). A study of Ghanaian women with cervical cancer reported an increase in five-years survival rate from 41% among all patients to 86.7% in women treated with radical radiotherapy as compared to palliative radiotherapy (23).

In SSA, the opportunities for specialty training in gynecologic oncology are rare. There are few trained local specialists who are qualified to train medical residents and fellows. At present, by our count, there are only seven SSA countries (including Ghana) in which a gynecologic fellowship training is currently offered. Most of these programs are very recent, with the oldest program, in South Africa, only dating back to 2008. Brain drain further depletes local human capacity as foreign-trained physicians remain in high-income countries to practice (24). Existing facilities are over-utilized, and because patient care is prioritized, the time allotted to education and training programs is sometimes cut short. LMICs are also limited by surgical supplies, imaging, anesthesia capacity, radiation equipment, and chemotherapeutics.

## Importance of Global Partnerships in Healthcare Capacity Building in Sub-Saharan Africa

Strengthening global health in Africa, in many cases, makes use of partnerships that were originally established to battle communicable diseases such as tuberculosis and HIV. Global collaborations are based on many motivations, including the link between health and economic development, the belief in a humanitarian agenda, and the recognition that healthcare developments can improve well-being globally (25).

Partnerships between institutions in LMICs and high-income countries can utilize infrastructure and relationships to access funding and build healthcare and research capacity in a part of the world that is disproportionately burdened with gynecological cancers and has limited access to resources and care (21, 26). Examples of partnerships in LMICs include the development of a training curriculum for OBGYN resident physicians in Central America based on guidelines from the Council for Resident Education in Obstetrics and Gynecology (27) and a collaboration between Health Volunteers Overseas (HVO), the Society of Gynecologic Oncology (SGO), and the American Society of Clinical Oncology to provide surgical training in Vietnam and Honduras (28). Similar programs have been established in Africa, including a partnership between the Botswana Harvard AIDS Initiative Partnership and the Beth Israel Deaconess Medical Center to provide eight weeks of gynecologic oncology services in Botswana (26), and a partnership between the University of Toronto and the Academic Model To Provide Healthcare to develop cervical cancer screening and gynecologic oncology programs at Moi University in Kenya. Technology and virtual relationships have been utilized by the University of Birmingham and Nigerian pathologists, who are providing training over Skype (29).

Many existing partnerships in gynecologic oncology care are based on a model of providing periodic assistance and training. Most are relatively new, utilize already established teaching hospitals and medical schools, require outside funding, and rely on external mentorship. Chuang et al. outlines 11 international professional organizations that are well-suited to provide training and education for healthcare providers that care for women with gynecological malignancies in LMICs. One such association is the International Gynecologic Cancer Society, which seeks to improve the quality of care women with gynecological cancers receive through education and training. The Gynecologic Oncology Global Curriculum & Mentorship Program is designed to facilitate connections between high-income country resources and LMICs seeking gynecologic oncology training. There is significant room for additional collaborations based on existing resources. In particular, academic institutions are ideal global partners given their expertise in clinical training, research infrastructure, and commitment to education.

### Gynecologic Oncology Training Programs Across Sub-Saharan Africa

Several gynecologic oncology fellowships have been established across the African continent. These programs share qualities such as global collaboration with international mentors, research components, and association with a teaching hospital (Table 1) (30). Several of the fellowship programs arose from cervical cancer screening initiatives that leveraged international collaborations and funding (Table 1).

**Table 1:** Gynecological oncology fellowship and training programs in Sub-Saharan Africa.

**Gynecological oncology fellowship programs**					
**Country / facility**	**Year start / stop**	**Global partnerships**	**Fellowship structure**	**Certifying body**	**Currently training /graduated**
**Ethiopia /** Black Lion Hospital (BLH)	2013	Initial HIC Partnership with Emory University and University of Toronto Subsequent HIC Partnership with Emory University and University of Michigan, joined by Martin Luther University*, subsequently joined by International Gynecologic Cancer Society (IGCS) in 2017	3 yr. program, local mentorship from practicing gynecologic oncologists (30–32)	Addis Ababa University The Association of Gynecological Oncology, Germany (AGO)−2019	1 / 6 (BLH Program) 4 graduated in 2017 2 graduated in 2019 with AGO certification
**Ethiopia /** St. Paul's Hospital Millennium Medical College (SPHMMC)	2015	University of Minnesota, University of Michigan, The Association of Gynecological Oncology, Germany (AGO)*, subsequently joined by IGCS in 2017	2 yr. program, mentorship from HIC programs (30–32)	St. Paul's Hospital Millennium Medical College	2/ 3 (SPHMMC Program)
**Ethiopia /** BLH & SPHMMC	2017	IGCS, (International Mentorship provided directly through IGCS)**+**	2- 3 yr. program, mentorship provided directly from HIC oncologists, 1 local supervisor	IGCS independently certifies its graduates	5/0
**Ghana**/ Komfo Anokye Teaching Hospital	2013	University of Michigan	4 yr. program, mentorship from HIC program and local mentorship from 1 practicing gyn-oncologist	Ghana College of Physicians and Surgeons	2 /3
**Kenya** / Moi University Medical School and Moi Teaching & Referral Hospital	2012	Academic Model Providing Access to Healthcare (AMPATH), University of Toronto, Society of Gynecologic Oncology of Canada, subsequently joined by IGCS in 2017	2 yr. program, HIC mentorship support for first 2 years, then local mentors took over (30, 33)	Moi University	0/7**
	2017	IGCS (International Mentorship provided directly through IGCS)**+**	2–3 yr. program, 2 HIC mentor from HIC programs, 2 local supervisors	IGCS independently certifies its graduates	3 / 1
**Mozambique** / Hospital Central de Maputo	2017	IGCS (International Mentorship provided directly through IGCS)**+**	2–3 yr. program, 2 local gynecology mentors, 5 HIC mentors, away rotations in Brazil for trainees (34)	IGCS independently certifies its graduates	3 / 0
**South Africa** / University of Cape Town, University of Pretoria (UP), University of Stellenbosch	2008	The South African Society of Gynaecologic Oncology joined with IGCS as part of strategic alliance partnership in 2020***	2 yr. program, local mentorship from practicing gyn-oncologists (30)	Health Professions Council of South Africa, Medical and Dental Professions Board	4 / NA (UP)
**Uganda** / Uganda Cancer Institute (UCI) and Mulago National Referral Hospital	2018	IGCS (International Mentorship provided directly through IGCS)**+**	2–3 yr. program, 2 HIC mentors and 1 local supervisor (30, 34)	IGCS independently certifies its graduates	5 / 2
**Zambia** / University Teaching Hospital—Women and Newborn	2018	IGCS (International Mentorship provided directly through IGCS)**+**	2–3 yr. program, 3 HIC mentors and 2 local supervisors (30, 35)	IGCS independently certifies its graduates	1/ 0
**Gynecological oncology training programs**					
**Botswana /** Scottish Livingstone Hospital	2017	Botswana Harvard AIDS Initiative Partnership, Beth Israel Deaconess Medical Center	2-weeks pilot+, HIC mentorship support (26)++	NA	NA
**Malawi /** Kamuzu Central Hospital	2015	University of North Carolina Project Melawi	1-week pilot project, surgical oncology, HIC mentorship support (36)	NA	NA / 1

* *Information regarding Ethiopia programs verified by Dr. Dawit Worku, MD, OBGYN, Assistant Professor Gynecologic Oncologist, Addis Ababa University*.

** *Kenya program information verified by Barry P Rosen, MD, Gynecologic Oncologist, Beaumont Gynecologic Oncology Associates*.

+ *IGCS program information verified by Susan Ralph, IGCS Mentorship and Training Program Manager via conference call*.

*** *Information from https://igcs.org/welcome-sasgo/*.

++ *Botswana program information verified by Rebecca Luckett MD, MPH, OBGYN, Associated Physicians of Harvard Medical Faculty Physicians at Beth Israel Deaconess Medical Center, Inc*.

## Gynecologic Oncology Fellowship at the Komfo Anokye Teaching Hospital, Ghana

### History

An OBGYN residency program was established in Ghana in 1989 through collaboration between Ghanaian academic hospitals and the University of Michigan, with funding from the Carnegie Corporation. To date, 246 OBGYN resident physicians have been trained, of which 236 (95.9%) certified specialists have remained in-country (24, 37–39). Subsequently, the University of Michigan has continued long-standing relationships with Ghanaian medical schools (40–42) and has been committed to an ongoing exchange of faculty and trainees (43). Building upon the successful OBGYN residency programs, the GCPS developed subspecialty training programs to further improve clinical care and promote research in certain specialized areas. These include fellowships in Maternal-Fetal Medicine, Reproductive Health and Family Planning, Urogynecology, and Gynecologic Oncology.

### Goals

The gynecologic oncology fellowship was designed to produce well-rounded gynecologic oncology subspecialists. Graduates of the fellowship are expected to provide clinical care, conduct research, train future fellows, and provide advice, leadership, and policy guidance related to gynecologic oncology (44).

### Entrance Requirements

Entrance requirements include passing a selection interview, completion of a residency in obstetrics and gynecology, membership or fellowship in the GCPS, the West Africa College of Surgeons, or other accredited or recognized colleges. Applicants who hold a membership in the GCPS will complete a total of four years of clinical training (two years for general gynecology and other rotations, and two years of gynecologic oncology). Those who hold fellowship will do two years of clinical training in gynecologic oncology (44).

### Program Structure

The fellowship lasts a total of four years. Clinical training is composed of rotations within gynecologic oncology and related specialties. The 1st and 2nd year of training focus on core medical and surgical training on gynecologic oncology. Fellows also manage some general gynecology patients, particularly those who need complex pelvic surgery. The third year of training includes one- to two-months rotations in various related subfields, including radiation and medical oncology, pathology, general surgery, urology, plastic surgery, and wound care. During this time, fellows continue their core gynecologic oncology training, while spending several days a week on these related rotations. Days spent on related rotations are focused on inpatient management, surgical care, and emergency care. Time spent with medical oncology and radiation oncology is focused on management of patients in clinic. The fourth and final year of training consists of a six-months external rotation in a department of gynecologic oncology in a training center outside of Ghana. During the final six months of the program, fellows complete and defend their dissertation and take their certifying board exam. To supplement training conducted in Ghana and the six-months external rotation, the fellowship program also has a designated outside faculty mentor from the University of Michigan who periodically comes to do hands-on teaching in Ghana each year.

### Training Sites

All clinical training in Ghana is done at the Komfo Anokye Teaching Hospital (KATH), a tertiary hospital in Ghana's second largest city, Kumasi (44). Trainees can elect to undergo a six-months external rotation in another country in order to have exposure to additional diagnostic and therapeutic training.

### Education

Fellows gain experience observing and performing a wide range of surgical procedures and post-operative care. In addition to hands-on clinical training, fellows attend and present at teaching sessions and monthly tumor boards, conduct research, write reports, and attend clinical rounds, meetings, and journal clubs.

### Syllabus/Curriculum

The curriculum was developed in collaboration with Ghanaian and University of Michigan faculty. The structure was adapted from gynecologist oncology training conducted in Ethiopia. The curriculum was approved by the Ghana College of Physicians and Surgeons.

The fellowship includes comprehensive training in a wide variety of topics (Table 2). Fellows are trained to diagnose and provide pre- and post-operative care associated with the management of a wide range of gynecologic oncologic diseases. Surgical training involves not only procedures related to female reproductive organs but also the gastrointestinal tract and urinary tract. Related procedures, including cystoscopy, thoracentesis, bowel resection, and lymph node dissection, are also learned. Development of these clinical and surgical skills is the central focus of each year of fellowship training. Daily rounds are conducted with faculty, fellows, and residents. Weekly schedules consist of the following: Monday: clinic; Tuesday: procedures including exam under anesthesia, biopsies, colposcopy, and pap smears; Wednesday: inpatient management with expanded rounds; Thursday: major surgeries; Friday: didactics.

**Table 2:** Syllabus content for Ghana gynecologic oncology fellowship training program.

**Syllabus content**
• Basic gynecologic oncology
• General surgery techniques
• Cancer of the ovary
• Cancer of the uterus
• Cancer of the cervix
• Cancer of the vulva
• Cancer of the vagina
• Gestational trophoblastic diseases
• Medical oncology
• Radiation oncology
• Imaging in gynecological oncology
• Pathology in gynecological oncology
• Urology
• Bowel surgery
• Plastic surgery
• Wound care

A key part of oncology training for gynecologists is better understanding of radiation therapy and chemotherapy. A rotation in radiation oncology covers the mechanisms of radiobiology, building a treatment plan that includes radiation treatments, and managing subsequent complications. Spending time in the clinic with medical oncologists gives fellows the opportunity to better understand the basic biology of chemotherapeutic agents as well as the clinical uses and side effects. These rotations occur in the third year of the fellowship. While fellows do not need to be able to work completely independently in these areas, they should become comfortable with consulting and working with radiation and medical oncologists (44).

Didactic instruction is provided on the basic biology of carcinogenesis, tumor genetics, and cancer immunology. Fellows will gain understanding in the pharmacological characteristics of drugs used for the treatment of various disease states, and how to manage complications that may arise from treatment. Additional instruction is provided on the basics of pathology, imaging, epidemiology, biostatistics, and palliative care. This didactic education is provided through several forums. Monthly journal clubs are held with fellows and Ghanaian faculty. Weekly virtual teaching sessions are held with fellows and a University of Michigan faculty member. Monthly virtual tumor boards are conducted with involvement from fellows, pathologists, and gynecologic oncology faculty from KATH and the University of Michigan.

### Research Requirements

Fellows choose research topics within the first six months of the program and can work on their projects over the course of their training. Fellows consult with the department's statisticians, and research proposals are approved by their faculty mentor (44). The fellows are required to submit their dissertation six months prior to taking their final exams and are encouraged to publish after their exams. Submission and successful defense of a dissertation on a locally relevant gynecologic oncology topic is a requirement for graduation. In addition, fellows collaborate with Ghanaian and University of Michigan mentors to conduct and publish research. To date, the graduated fellow and three current fellows have a total of 31 publications in peer-reviewed journals, one-quarter of which were in collaboration with University of Michigan faculty. Research goals include both the generation of data that improves local and regional clinical care, as well as the development of fellows' careers in academic medicine.

### Evaluation

Fellows record their surgical procedures in a logbook, which is assessed regularly by their mentor to ensure that each fellow is adequately progressing in skills development. Fellows undergo quarterly assessments to ensure they are meeting milestones. Final examinations consist of an oral exam, written exam, and a defense of their research dissertation. During the oral exam, fellows are quizzed for one h on gynecologic oncology topics. The written exam is comprised of 100 multiple-choice questions. The defense consists of a 15-minute PowerPoint presentation by the fellow, followed by a 45-minute defense. Once a fellow passes this examination, they are awarded the fellowship (44).

### Local and External Faculty Support

Primary mentors for the fellows are specialists within the Department of Obstetrics and Gynecology at KATH. Additionally, radiation oncologists, medical oncologists, and pathologists are made available for fellows to consult and train with throughout the program. Furthermore, fellows benefit from instruction from external faculty. Annual surgical teaching on advanced techniques is done in Ghana by a visiting University of Michigan faculty member, Dr. Carolyn Johnston. Weekly teaching, review of readings, and presentations of case records is carried out virtually with Dr. Carolyn Johnston. A multidisciplinary tumor board is held monthly by gynecologic oncologists, pathologists, and radiation oncologists from both Ghana and Michigan. Drs. Richard Lieberman and Shruti Jolly, of Michigan Medicine Pathology and Radiation Oncology departments respectively, have not only participated regularly in monthly tumor boards but also taught at KATH and provided ad lib remote consultations.

Regarding local sustainability of the fellowship, the graduation of a fellow in 2016 was a key step in ensuring capacity for continued local training. This first graduate of the fellowship remains at KATH as a gynecologic oncology faculty member, and he oversees clinical and surgical training of current fellows. The University of Michigan has decades of partnership with academic medical institutions in Ghana, and is committed to continuing partnerships in gynecologic oncology. Collaborations in research and through the monthly inter-institution tumor board are beneficial to trainees at both KATH and the University of Michigan.

The COVID-19 pandemic has challenged traditional models of global partnerships, with safety concerns about international visits from partners to provide local surgical training. With continued advancement in surgical techniques, including laparoscopy and new methods of lymph node sampling, there is continued benefit from University of Michigan faculty providing periodic surgical training when international travel is safe. Virtual engagement between the KATH fellows and University of Michigan faculty, including virtual weekly teaching sessions, monthly tumor boards, and virtual collaboration on research, serve as important models of sustainable collaboration.

## Reflections From Gynecologic Oncology Fellows in Ghana

To gain a personal perspective on gynecologic oncology fellowship training, two physicians involved currently and previously involved in the KATH gynecologic oncology fellowship completed in-depth semi-structured interviews. The first interviewee is a current fellow scheduled to graduate in 2020. The second interviewee graduated from the fellowship in 2016 and is now a local mentor for current fellows in the program. Questions focused on their perspectives about the program; its strengths and its weaknesses, and challenges in the delivery of gynecological oncology care in Ghana. See Table 3 for representative quotations.

**Table 3:** Representative quotations from qualitative interviews with past and current fellows.

**Motivation**	
	*“When I was doing OBGYN, I also realized that for a certain group of women with cancer, there were very few people taking care of them.”*
	*“There are not a lot of gyn-oncologists, in Ghana. So, I think that there are a lot of patients here that will need me. I will be [more] use in Ghana than going elsewhere, where there are lots of doctors and all those fancy things. I think that when I when I'm in Ghana, then I'll be able to help the people who need me most.”*
	*“I see the cases that she [Dr. Carolyn Johnston, University of Michigan] does and there are some cases that previously we would have done and said this is not operable. But when she comes to take four or five hours and it becomes operable. I realized that we needed to do more.”*
**Strengths of the Program**	
	*“We have every kind of gynecology oncology case you can think of in Ghana.”*
	*“In fact, we have too many [cases]. The problem we have now is time to operate.”*
**Challenges of the Program**	
	*“We don't have a lot of instruments for surgeries. …You have to improvise, especially with retraction. We really are in need of those retractors.”*
	*“We had a lot of backlog, even before COVID. So as soon as we start operating, some of the backlogged patients will have been dead already.”*
	*“I need more people to be interested in gynecology oncology. We need more fellows.”*
	*“Laparoscopic surgeries…That is what we lack most. And that is one area that if I get any opportunity, I will gladly grab it. Because, it is the way the world is moving.”*
	*“Screening for cervical cancer is <5% in Ghana.”*
	*“The [radiation] machines are broken down. Also,… the chemotherapy drugs are expensive. Some of them are not on the national health insurance. So, at times, the patients have to buy and because patient cannot buy, the patient tends to default. And that's also causing a lot of problems for us.”*
**Value of Global Partnerships**	
	*“I think the University of Michigan has been so much of a help to Ghana, to my hospital, and to me personally. They've been so fantastic to us and [what] I want to tell the University of Michigan is that they are really, really saving lives in Ghana and that they should keep it up. They are really helping humanity.”*
	*“Because we can train fellows in Ghana, women with cancers are going to have quality care in Ghana. Instead of having to rely on people coming from outside to take care of them… It is because of this fellowship that was a collaboration with the University of Michigan and the Ghana College.”*

Primary motivations for applying for the gynecologic oncology fellowship were a genuine desire to help improve care for Ghanaian women, a personal connection with gynecological cancers, and strong local and international mentors in gynecologic oncology. Fellows felt that strengths of their training program were wide exposure to various gynecologic cancers, as well as a high volume of surgical cases. Challenges of the fellowship include limitations in surgical equipment and operating room time in Ghana, a lack of access to frozen section pathology, long waiting times for pathology results, and limited capacity for radiation and chemotherapy. Looking forward to the future of gynecologic oncology training in Ghana, concern was expressed about low numbers of future fellowship candidates and the desire for exposure to laparoscopic techniques. Fellows' concerns regarding the challenges in delivery of gynecologic oncology care to women in Ghana include lack of screening and early prevention programs, poor patient education, and lack of financial resources.

Fellows recognize the strength of the Michigan-Ghana partnership and its impact on training and OBGYN care in Ghana. Benefits include surgical and didactic mentoring, research partnerships formed with faculty and trainees, and involvement in a collaborative tumor board. Room for improvement includes regulations that preclude hands-on surgical training during elective time in Michigan.

## Conclusions

Gynecological malignancies are important contributors to morbidity and mortality worldwide and have a disproportionate burden in LMIC. The availability of trained gynecological oncologists is essential to provide screening, diagnosis, surgical care, and coordinate chemotherapy and radiotherapy. Gynecological oncology fellowship/training programs in sub-Saharan Africa, many developed as part of sustained global partnerships, are relatively new but are already having a positive impact. The gynecological oncology fellowship at KATH in Ghana is a four-years program developed in collaboration with the University of Michigan. The fellowship involves weekly virtual mentorship sessions, monthly tumor board meetings, in-country surgical training, an away observership and opportunities for research. Challenges of the fellowship include limited availability of surgical instruments, advanced technology including laparoscopy, affordable imaging, and timely pathology diagnoses. Among the program's successes, however, are the increasing number of patients managed for gynecologic malignancies, and an increasing number of complex pelvic surgical procedures that can be done in-country. Fellows are also advocates for patient education and screening in medical associations and the local media. The first gynecologic oncologist graduated from the KATH fellowship, and now serves as a mentor to future trainees.

The manuscript contributes to the literature by outlining the importance of global partnerships in high-impact subspecialized care like gynecologic oncology, describing the structure and commonalities of existing gynecologic oncology training programs across Africa, and discussing a gynecologic oncology fellowship program at KATH in Ghana. Details about the structure and content of the fellowship program at KATH can be utilized by other academic institutions to adopt similar fellowship programs. Limitations include the in-depth focus on a single fellowship, and the newness of the fellowship, which limits evaluation of longer-term impact. Further research is needed to evaluate quality improvements in clinical practice as a result of fellowship graduates, and to evaluate the sustainability and changes in the fellowship over time. In conclusion, by using the KATH fellowship as a model, the expansion of gynecological oncology training programs in LMICs is essential to improve access to life-saving gynecological oncology care.

## Author Contributions

AE was responsible for the literature search, drafting the original manuscript, and conducting interviews. AA-K and TK provided guidance, reviewed, and edited the manuscript. SB reviewed and edited the manuscript. TJ reviewed the manuscript. CJ reviewed and edited the manuscript. EL provided guidance, edited, and wrote portions of the manuscript. KA-A and AT proofread and edited the manuscript. All authors contributed to the article and approved the submitted version.

## Conflict of Interest

The authors declare that the research was conducted in the absence of any commercial or financial relationships that could be construed as a potential conflict of interest.
